# Determining the methodological rigor and overall quality of out-of-hospital clinical practice guidelines: a scoping review

**DOI:** 10.1186/s13049-025-01344-z

**Published:** 2025-02-21

**Authors:** Brendan V. Schultz, Timothy H. Barker, Emma Bosley, Zachary Munn

**Affiliations:** 1 JBI, School of Public Health, Faculty of Health and Medical Science, The University of Adelaide, Adelaide, SA Australia; 2 Queensland Ambulance Service, Department of Health, Queensland Government, 125 Kedron Park Road, Kedron, Brisbane, QLD 4031 Australia; 3 Health Evidence Synthesis, Recommendations, and Impact (HESRI), School of Public Health, Faculty of Health and Medical Science, The University of Adelaide, Adelaide, SA Australia; 4 School of Clinical Sciences, Faculty of Health, Queensland University of Technology, Brisbane, QLD Australia

**Keywords:** Clinical practice guideline, Ambulance, Guideline appraisal, Paramedicine, Pre-hospital

## Abstract

**Objectives:**

Out-of-hospital clinical practice guidelines (CPGs) guide paramedics, emergency medical technicians and first responders, but their quality remains uncertain. This scoping review aims to identify, aggregate and describe all literature that has used a structured appraisal instrument to assess the methodological rigor and overall quality of out-of-hospital CPGs.

**Methods:**

This study was conducted in accordance with the JBI methodology for scoping reviews and involved systematically searching the following databases and/or information sources with no publication or language limit applied: MEDLINE (Ovid), Embase (Elsevier), CINAHL with full text (EBSCO), Scopus (Elsevier), ProQuest Central (ProQuest).

**Results:**

This review identified 15 articles that appraised 311 unique out-of-hospital CPGs. These CPGs ranged in date of publication from 1998 to 2022. The majority of CPGs (267/311) were assessed using the Appraisal of Guidelines for Research & Evaluation (AGREE-II) instrument, with 146 guidelines appraised against two tools. Following aggregation, CPGs scored highest in Domain 4 (clarity of presentation) at 77.7% (SD = 15.1%), and lowest in Domain 5 (applicability) at 42.6% (SD = 23.7%). The average Domain 3 score (rigor of development) was 55.6% (SD = 25.7%). Of CPGs appraised against the AGREE-II instrument, 34.4% met our a priori definition of being high-quality (Domain 3 score of equal to or greater than 75%), while 31.3% were deemed medium-quality (Domain 3 score between 74% and 50%), and 34.3% were considered low-quality (Domain 3 score less than 50%). There were no significant changes observed in the average Domain 3 score over time (*p* = 0.092). 146 CPGs were assessed against the National Academy of Medicine criteria with 34.9% meeting all elements indicative of being a high-quality guideline, while 39 CPGs were assessed the 2016 National Health and Medical Research Council Standards for Guidelines with 0% meeting all criteria.

**Conclusions:**

Out-of-hospital CPGs currently have poor methodological rigor and are of medium to low overall quality. These results should be used to inform future research and initiatives that aim to standardize the methods used to develop guidelines used in this healthcare setting.

**Supplementary Information:**

The online version contains supplemesupplementary material available at 10.1186/s13049-025-01344-z.

## Background

Clinical Practice Guidelines (CPGs) are evidence-based statements designed to assist health professionals, patients, and policy makers to make informed clinical decisions [1, 2]. Briefly, when developed using rigorous methods these documents contain actionable recommendations that have transparently and judiciously considered the best available evidence balanced against evidence-informed considerations regarding economics and environmental impact and the opinion of experts and relevant stakeholders [3–5]. CPGs are ubiquitous within the provision of modern healthcare and can conceptually be considered an adjunct that bridges the chasm between evidence and clinical practice. CPGs are not always developed using rigorous, transparent, and robust methodologies and it has been postulated that up to 50% of current guidelines do not adhere to accepted standards or score poorly when assessed against structured appraisal instruments [6, 7].

CPGs are heavily utilized in the out-of-hospital environment by paramedics, emergency medical technicians and first responders to inform practice and supplement clinical decision-making. In recent decades, clinicians operating in this healthcare setting have transitioned from essential ‘stretcher-bearers’, tasked with providing simple care prior to arriving at hospital, to registered health professionals who can autonomously deliver interventions without transferring the patient to an emergency department [8, 9]. As the clinical cares provided by out-of-hospital healthcare providers are guided, directed, and informed by these CPGs, it is imperative that these documents are high-quality, and evidence based. Poorly developed CPGs represent an inherent patient safety risk as they may be influenced by undue biases, impracticable to follow or may lack external validity [10, 11]. Similarly, CPGs produced using nonempirical methods or approaches may recommend treatment that is inferior, infective, or even harmful [12–14].

There is an emerging body of evidence that have used various appraisal instruments to assess the methodological rigor and overall quality of out-of-hospital CPGs. Briefly, these studies have to date been limited to appraisals of CPGs from specific geographical regions or individual medical presentations [15–17]. As a result, the current state of out-of-hospital CPGs has not been described comprehensively, with this being identified as a priority area requiring further investigation in a recent paramedic research agenda [18]. It is anticipated that the outcomes of this review will map all available evidence and identify gaps for future research.

## Methods

This review was performed in accordance with the JBI methodology for scoping reviews and has been reported to conform with Preferred Reporting Items for Systematic reviews and Meta-Analyses extension for Scoping Reviews (PRISMA-ScR) [19, 20]. The overarching objective of this review was to answer the following clinical question: ‘what is the methodological rigor and overall quality of CPGs developed specifically for the out-of-hospital setting where assessed using a structured appraisal instrument?’ Prior to commencement, a detailed a priori protocol was developed outlining the intended methodology and the population, concept, and context this scoping review would address [21].

### Search strategy

To locate all published studies relevant to the review question, we developed a search strategy in consultation with an Information Scientist. This involved performing a preliminary unstructured search of MEDLINE (Ovid) and EMBASE (Elsevier) to identify articles relevant to the topic. The medical subject headings, keywords, and Emtree terms of these articles were used to inform the full search strategy that was adapted for each database and/or information source as required. We conducted a systematic search of Embase, CINAHL, Scopus and ProQuest Central on April 3rd, 2024. The search strategy used has been provided as Appendix 1. We included all types of studies (primary and secondary) that assessed an out-of-hospital CPG using a structured appraisal instrument. To ensure the concept was comprehensively examined, no language or publication date limits were applied.

### Study selection and screening

Following the removal of duplicates, the titles and abstracts of records were screened independently by two reviewers using Covidence (Veritas Health Innovation, Melbourne, Australia), with disagreements resolved by discussion or with the involvement of an independent third reviewer. Potentially relevant records were then retrieved as a full-text and assessed in detail against the eligibility criteria by the same two independent reviewers.

In this review, we defined a structured appraisal instrument as any tool that uses fixed criteria to assess one or more of the following aspects of a CPG to produce an overall score: (i) methodological rigor and transparency of development; (ii) involvement of stakeholders; (iii) applicability into clinical practice. A CPG was defined as any document that contained clear treatment recommendations that guide, direct, or inform policy or clinical practice. Literature was excluded if it appraised CPGs that were designed for other clinical settings such as in-hospital (emergency departments, critical care units, or surgical wards) or military and/or combat environments. Studies were also excluded if they appraised documents other than CPGs such as clinical algorithms, treatment protocols or care pathways. Studies that appraised both out-of-hospital and in-hospital CPGs were included, however, only data on out-of-hospital specific guidelines were extracted and subsequently analyzed.

### Data extraction and coding

Data was subsequently extracted for all articles meeting the inclusion criteria by two reviewers independently using an extraction tool created in Microsoft Excel (Microsoft Corporation, Washington, United States). Data extraction was performed as a two-phase process with information retrieved from both the primary article, and for each individual CPG the study had appraised. To ensure all relevant items were collected, the extraction tool was first piloted on three sources. Between publication of the initial study protocol and completion of the data extraction process, the following amendments were made to the extraction tool: (a) ‘evidence grading classification used’ was changed to ‘Grading of Recommendations, Assessment, Development and Evaluation (GRADE) approach used’; and (b) ‘guideline development method used (e.g., de novo, adapted, adopted)’ was removed. These changes were made secondary to the availability of this information in the included sources.

CPGs were not individually appraised by the reviewers during data extraction, rather the appraisal that had already been performed and reported within the study was retrieved. In instances that data could not be extracted, multiple attempts were made to contact the corresponding author. If no reply was received, this data was considered missing. Details regarding the attempts made to contact authors are described in Supplementary Material 1. CPGs were given a unique study identifier if they were reported in an anonymized format within the included studies. All reviewers collectively discussed anonymized CPGs to minimize the likelihood of duplicates. In this scoping review, the quality of included studies was not assessed, and no risk of bias assessment was performed [19]. The extraction tool and raw data reported in this study are provided as Supplementary Material 2.

### Data analysis and presentation

If not explicitly stated, CPGs were considered to originate from the country of the listed primary author with countries then categorized based on the World Health Organization regions. CPGs were categorized as being developed by the following umbrella classifications: (i) individual ambulance or emergency medical service (e.g. South Australia Ambulance Service, Queensland Ambulance Service, Alabama Emergency Medical Service); (ii) national emergency medical service group (e.g. Joint Royal Colleges Ambulance Liaison Committee, National Association of State EMS Officials, National Association of EMS Physicians); (iii) professional medical society (e.g., Stroke Foundation, Eastern Association for the Surgery of Trauma, American College of Medical Toxicology); (iv) academic group (e.g. group of individuals with no clear affiliation); and (v) national institute or governing body (e.g. National Institute for Health and Care Excellence, Scottish Intercollegiate Guidelines Network, American Heart Association).

To account for different appraisal instruments that were used, results were grouped based on similar characteristics but reported independently. In instances that multiple primary sources appraised the same CPG using the AGREE-II tool, the scores for each domain were aggregated together and calculated using the formula outlined in the instrument’s user manual [22]. This was performed on 29 individual CPGs, further information can be found in Supplementary Material 2. In circumstances where primary sources did not present the individual AGREE-II domain scores for each CPG, and instead provided a calculated total, the average of this total was reported as a surrogate. To determine the quality of CPGs assessed with the AGREE-II instrument we used the following threshold cutoffs: (i) high-quality - Domain 3 score equal to or greater than 75%; (ii) medium-quality - Domain 3 score between 74% and 50%; and (iii) low-quality - Domain 3 less than 50%. This threshold for high-quality was used to align with guidance provided by the authors of the AGREE-II tool [23]. CPGs appraised against the National Academy of Medicine (NAM) criteria, the 2016 National Health and Medical Research Council (NHMRC) Standards for Guidelines, and the JBI Critical Appraisal Checklist for Text and Opinion were reported as a count of when the ‘yes’ value was denoted.

Extracted data has been presented descriptively, with categorical variables reported as counts and percentages while continuous data was reported as mean (standard deviation). The Cochran-Armitage test was performed to determine if the methodological rigor of CPGs and use of the GRADE approach had changed over-time, while categorical variables were statistically analyzed using the Chi-Square test (χ2) and Fisher’s exact test, as appropriate. To determine if there was an association between the Domain 3 score and the group that produced the CPG, the Shapiro-Wilks W test was first performed to determine if the data was normally distributed. Subsequent analysis was performed using the non-parametric Kruskal-Wallis test with post-hoc comparisons made using Dunn’s method with Bonferroni correction. Due to the small sample size (*n* = 4), CPGs produced by national emergency medical service groups were excluded from post-hoc comparisons. All tests were two-sided with a *p* value less than 0.05 considered statistically significant. All statistical analysis was performed using SPSS for Windows (version 28.0, IBM, New York, United States).

## Results

### Search results

**Fig. 1:**
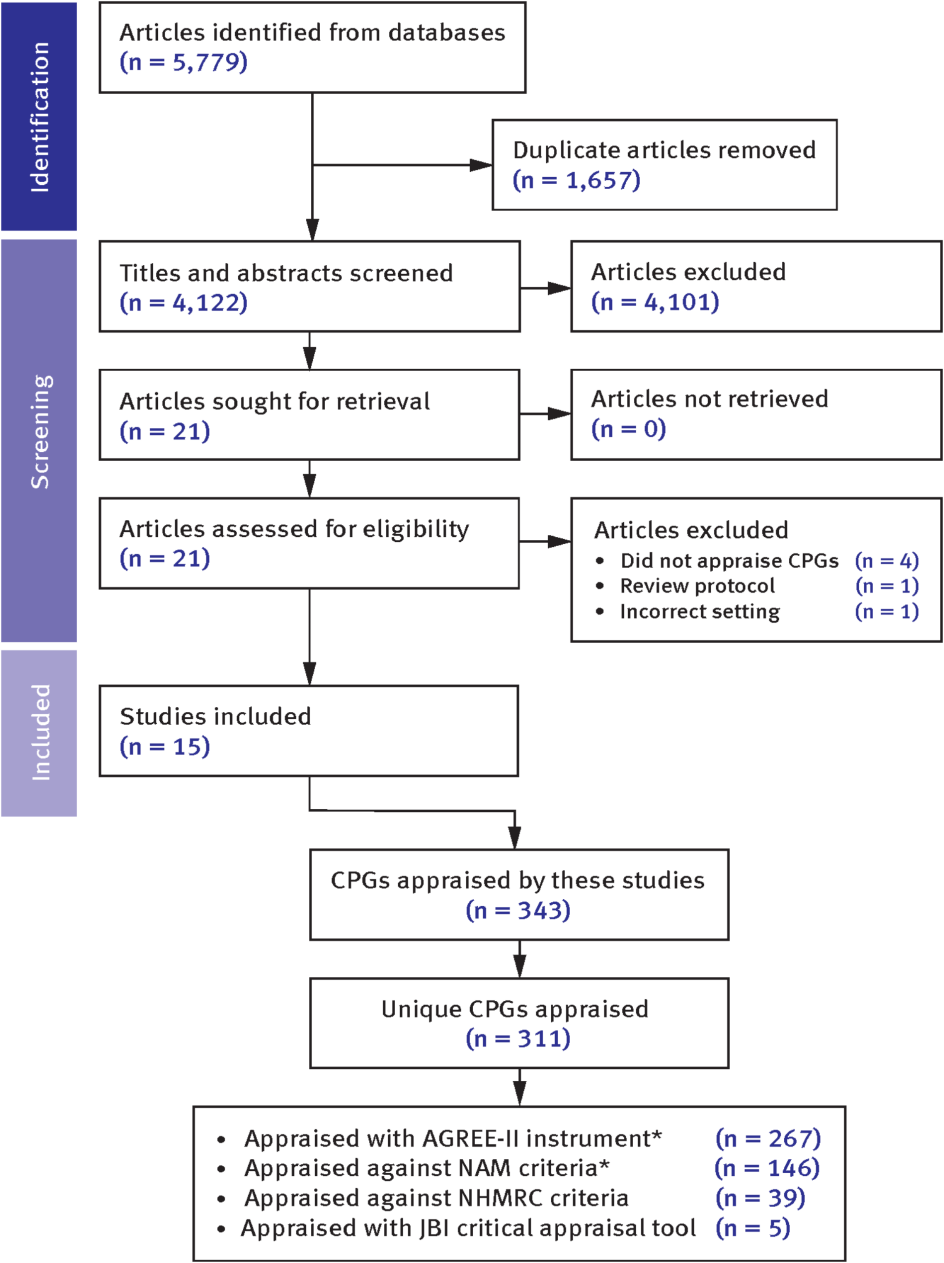
PRISMA flowchart. Figure 1 footnotes: *146 CPGs were appraised by both the AGREE-II instrument and against the NAM criteria

There were 5,779 records initially identified after performing the search strategy. Following the removal of duplicates, the titles and abstracts of 4,122 records were screened. 21 records were determined to be eligible for inclusion following this screening, and the full-text reports were retrieved for further screening against the eligibility criteria. The complete PRISMA flow diagram that depicts the flow of information through all phases of this review is displayed as Fig. 1. Following screening, a total of 6 reports were excluded for failing to meet the eligibility criteria, the reasons for exclusion were due to not appraising CPGs (*n* = 4); appraising CPGs used in a different setting (*n* = 1); and being a review protocol (*n* = 1) (the details of these articles has been provided in Supplementary Material 3). Therefore, the reports of 15 studies were eligible for inclusion within this scoping review [15–17, 24–35].

### Characteristics of included studies

The included studies were published between 2016 and 2023. The most common methodology used by to identify out-of-hospital CPGs was searching online databases and/or repositories 9/15 (60.0%), followed by targeted sampling methods whereby guidelines were requested from select organizations (20.0%), a hybrid approach of database searching and targeted sampling (13.3%), and manually retrieving publicly available CPGs from the websites of individual ambulance services (6.7%). The AGREE-II instrument was the main appraisal tool used in these studies to assess CPGs (73.3%), followed by a combination of AGREE-II and the NAM criteria (13.3%), the JBI Critical Appraisal Checklist for Text and Opinion (6.7%), and the 2016 NHMRC Standards for Guidelines (6.7%). The average number of out-of-hospital CPGs appraised by each study was 24.5 (SD = 25.24). Data was successfully extracted from 14 of the 15 studies that were included, with information not retrieved from one source as it was presented in an aggregated manner whereby the CPGs were reported in combination with algorithms, clinical protocols, and review documents [25].

### Characteristics of included CPGs

In total, 343 CPGs were appraised however following the removal of duplicates, this number was reduced to 311 unique guidelines, with 146 guidelines appraised by two tools (refer to Fig. 1). The CPGs included in this review provided guidance on 23 overarching medical topics, with resuscitation (23.5%), trauma (20.9%), obstetrics (13.8%) and pain management (10.3%) the dominant categories. In contrast, guidance on the management of stroke (cerebrovascular accident), palliative care, and seizures was contained in just 3.9%, 2.8% and 1.3% of CPGs that were appraised. Similarly, only 7.8% of CPGs were specific to pediatric patients. There was a small number of CPGs that addressed non-clinical aspects which included fatigue management (*n* = 1), medication safety (*n* = 1), and the management of bystanders (*n* = 1). CPGs ranged in publication date from 1992 to 2022, with 41.0% published between 1998 and 2016 and 59.0% published from 2017 onwards.

**Fig. 2:**
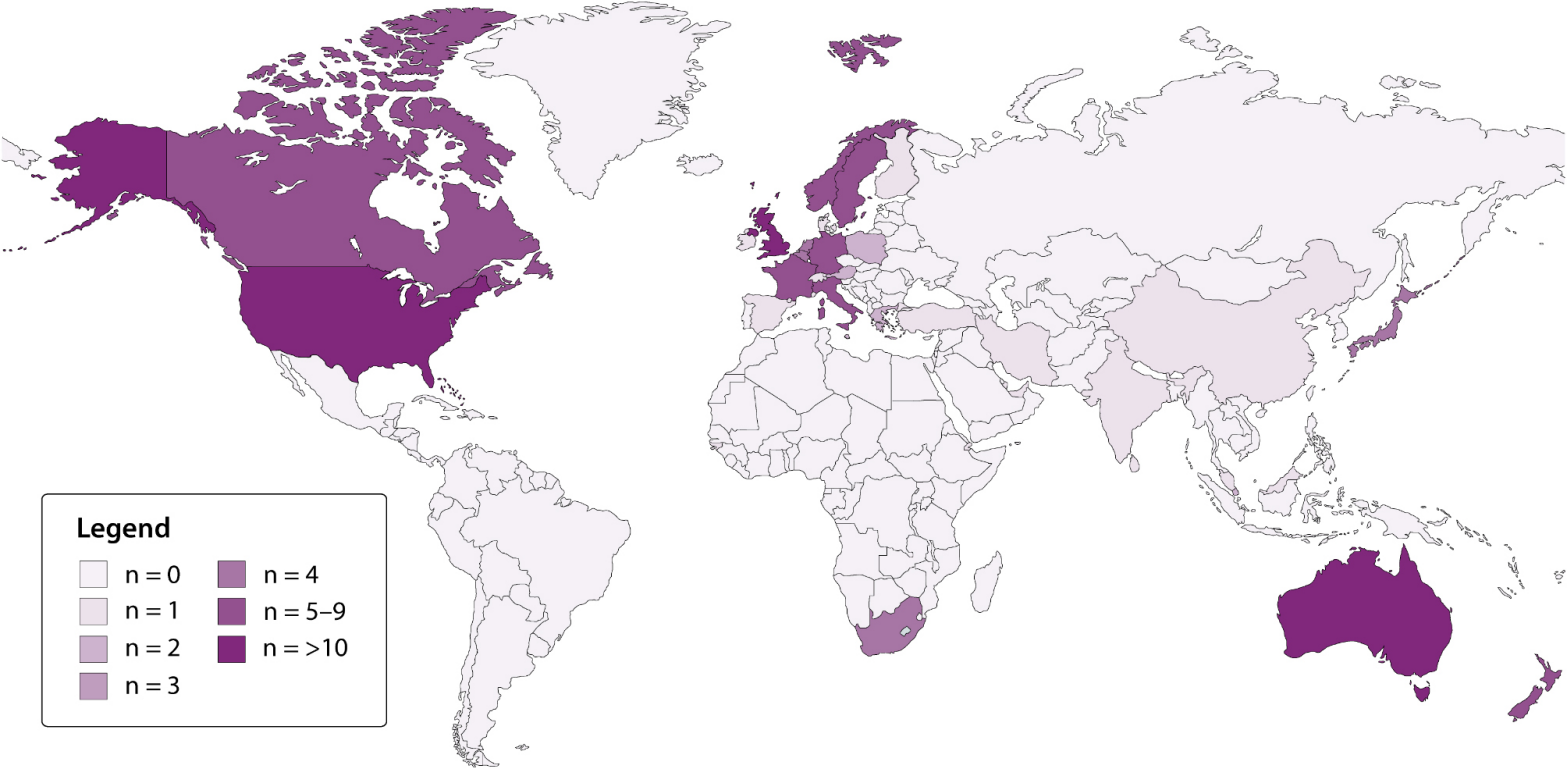
Number of CPGs developed by each individual country

CPGs were mainly developed by professional medical societies (129/311, 41.5%), while individual ambulance or emergency medical services, national institutes or governing bodies, academic groups, and national emergency medical service groups represented 27.0%, 22.5%, 7.4% and 1.6%, respectively. The GRADE approach was used in 23.5% of the CPGs that were appraised, with a significant increase in the use of this framework observed over time (*p* < 0.001). This methodology used exclusively by CPGs produced by professional medical societies and national institutes or governing bodies (Table 1). The majority of CPGs (98.1%) originated from the Region of the Americas, Western Pacific Region, and European Region. The number of CPGs developed by each individual country are geospatially presented in Fig. 2.

**Table 1:** Characteristics of appraised CPGs

Characteristics	*n* = 311 (%)
Guideline producer category	
Academic group	23 (7.4)
Individual ambulance/EMS	84 (27.0)
National EMS group	5 (1.6)
National health institute or governing body	70 (22.5)
Professional medical society	129 (41.5)
Year of publication^1^	
1998–2003	8 (2.7)
2004–2009	31 (10.3)
2010–2016	84 (28.0)
2017–2022	177 (59.0)
Target demographic of guideline	
Adults	140 (45.0)
All ages	147 (47.3)
Paediatrics	24 (7.7)
GRADE approach used	73 (23.5)
Guideline published in peer-reviewed journal	188 (60.4)

Table 1 footnotes: ^1^ Year of publication is unknown for 11 CPGs. EMS: emergency medical service; GRADE: Grading of Recommendations Assessment, Development and Evaluation

### Appraisal of included CPGs

Of the 311 unique CPGs that were appraised, 267 were assessed using the AGREE-II instrument [15–17, 24–33]. The average number of reviewers used in each study was 3 (SD = 1.75). Most CPGs were appraised across all domains (230/267, 86.1%), however 13.1% (*n* = 35) were appraised only on Domain 3 (rigor of development), and 0.8% (*n* = 2) were presented as a total combined score, per the methods of their corresponding primary articles [24, 27].

**Fig. 3:**
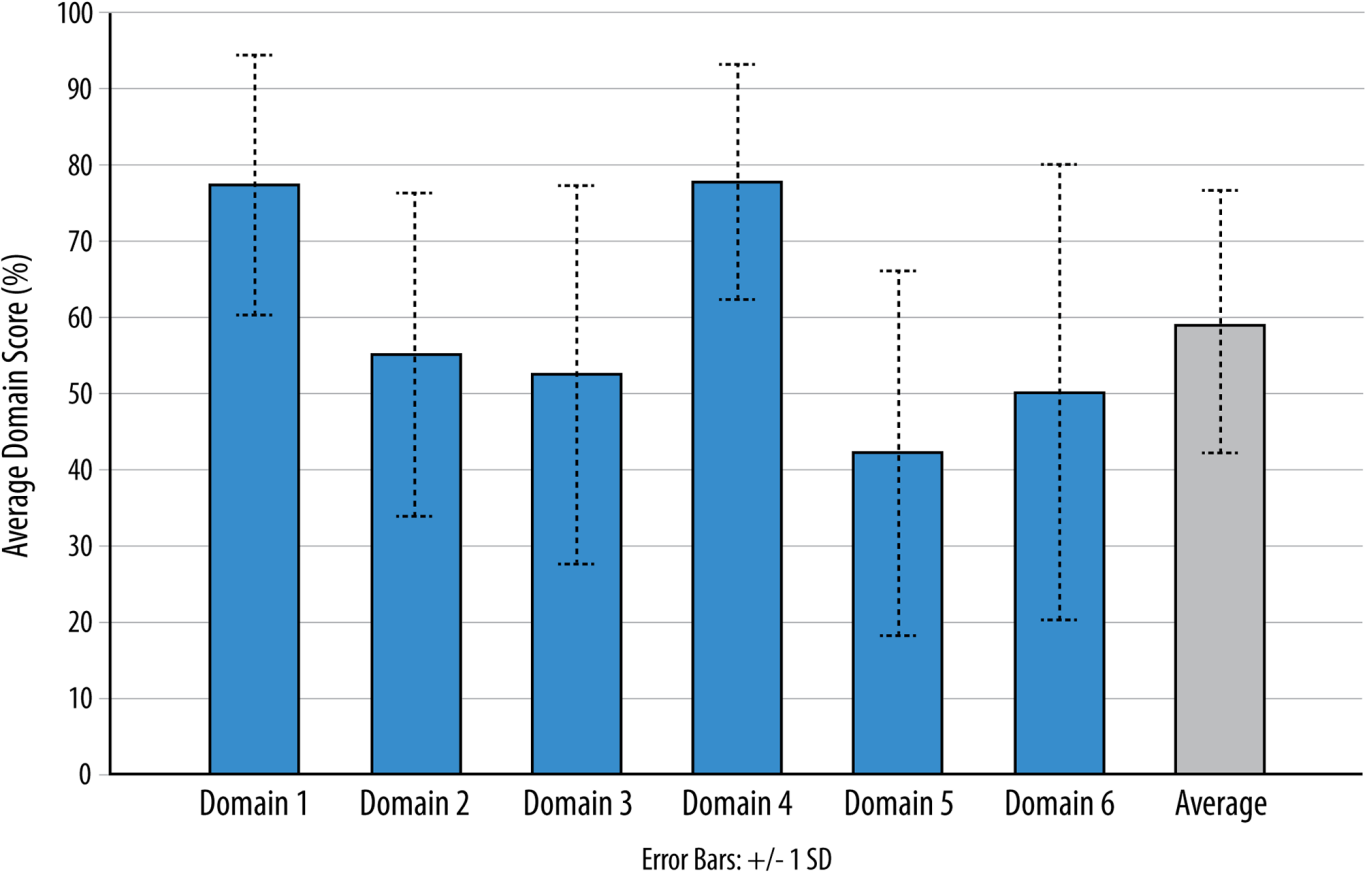
Average domain scores of CPGs appraised using the AGREE-II instrument. Figure 3 footnotes: SD: Standard deviation

Following aggregation, CPGs scored highest in Domain 4 (clarity of presentation) at 77.7% (SD = 15.1%), whereas the lowest score was seen in Domain 5 (applicability) at 42.6% (SD = 23.7%). The scores for other domains including a calculated average calculated of all domain scores are displayed in Fig. 3. The average Domain 3 score was 56.6% (SD = 25.7%), with no significant changes observed in the average score in this domain over time (*p* = 0.092). Overall, 91/265 (34.3%) of CPGs met our quality threshold of being a high-quality (score of equal to or greater 75% in Domain 3), while 31.3% were deemed medium-quality, and 34.3% were considered low-quality.

There was a significant association between the group that developed the out-of-hospital CPG and the observed domain 3 score [χ^2^ (4, *N* = 265) = 66.3, *p* < 0.001]. The mean score in this domain was 76.7% for CPGs produced by national institutes or governing bodies, 57.8% for professional medical societies, 49.2% for academic groups, 39.0% for national emergency medical service groups and 23.3% for individual ambulance or emergency medical services. Pairwise comparison between these groups is displayed in Table 2.

**Table 2:** Pairwise comparison of CPGs based on developing group category

Guideline producer category^1^	Domain 3 Score, mean (SD)	Individual ambulance/EMS, H, (*p*-value)^2^	Academic Group, H, (*p*-value)^2^	Professional medical society, H, (*p*-value)^2^	Meet all NAM criteria, *n*, (%)	Academic Group, *p*-value^4^	Professional medical society, *p*-value^4^
Individual ambulance /EMS	23.3 (23.3)	-	-	-	N/A^3^	-	-
Academic Group	49.2 (18.3)	50.2 (0.07)	-	-	0/20 (0)	-	-
Professional medical society	57.8 (21.7)	-79.9 **(< 0.001)**	-29.7 (0.49)	-	20/86 (23.3)	**< 0.001**	**-**
National health institute or governing body	76.7 (11.1)	-150.5 **(< 0.001)**	-100.3 **(< 0.001)**	70.6 **(< 0.001)**	31/40 (77.5)	**< 0.001**	**< 0.001**

Table 2 footnotes: ^1^ Due to the small sample size (n = 4 in Domain 3 score and n = 0 in NAM criteria), CPGs produced by national emergency medical service groups were excluded from post-hoc comparisons. ^2^ Analysis was performed using Kruskal-Wallis with post-hoc comparisons made using Dunn’s method with Bonferroni correction. ^3^ No CPGs produced by individual ambulance or emergency medical service were assessed against the NAM criteria. ^4^ Analysis was performed using Fisher’s exact test. SD: Standard deviation; EMS: /emergency medical service

The NAM (previously known as the Institute of Medicine) criteria was used to appraise 146 CPGs, with this this adapted from their publication entitled ‘Clinical Practice Guidelines We Can Trust’ [2]. This appraisal was conducted following an independent full-text review by two reviewers, with disagreements and final consensus determine by involvement of a third reviewer [27, 30]. When assessed against this tool, CPGs scored highly against the criteria of containing a description of benefits, harms, and alternate care options (142/146, 97.3%), providing a summary of evidence synthesis (86.3%), being developed, or endorsed by one or more professional organizations or associations (86.3%), and containing systematically developed recommendations (82.9%). Conversely, CPGs scored poorly in the criteria of providing a description of search strategy (54.8%), performing, or containing key elements of a systematic review of literature (52.1%), providing a description of study selection (47.9%), and synthesizing evidence (47.3%). Overall, only 34.9% of CPGs that were appraised against this tool met al.l the NAM criteria indicative of being a high-quality guideline. CPGs were significantly more likely to meet al.l NAM criteria if they were developed by a national institute or governing body, in comparison to a professional medical society or academic group (77.5% vs. 23.3% vs. 0%, [χ^2^ (2, *N* = 146) = 47.8, *p* < 0.001]) (Table 2).

There were 39 CPGs that were appraised against the 2016 NHMRC Standards for Guidelines, with these guidelines all originating from individual ambulance services in Australia. Briefly, this appraisal was undertaken as part of a content analysis of obstetric and neonatal CPGs with this performed collectively by all reviewers with disagreements resolved through consensus [34]. Of the 39 CPGs that were appraised, 0 (0%) met al.l nine NHRMC standards which are comprised of: (i) be relevant and useful for decision making; (ii) be transparent; (iii) be overseen by a guideline development group; (iv) identify and manage conflicts of interest; (v) be focused on health and related outcomes; (vi) be evidenced informed; (vii) make actionable recommendations; (viii) be up-to-date; and (ix) be accessible. Data on how each individual CPG scored against these standards was not able to be provided despite correspondence with the authors.

Only a small number of CPGs (*n* = 5) were appraised using the JBI Critical Appraisal Checklist for Text and Opinion, with these CPGs all being produced by individual ambulance services in the United Kingdom [35]. Across the six criteria of this tool, CPGs on average scored greater than or equal to 80% in the following elements: (i) is the source of opinion being clearly identified; (ii) does source of opinion having standing in the field of expertise; (iii) are the interests of the relevant population the central focus of the opinion; (iv) is there reference to extant literature; and (v) if any incongruence with the literature/sources is logically defended. CPGs however, had an average score of 20% in the criteiria of ‘Is the stated position the result of an anlaytical process?’, and ‘Is there logic in the opinion expressed?’. Overall, 3/5 of the individaul CPGs appraised met greater than or equal to 80% of the tools criteria, while the remaining CPGs met 60% and 40%, respectively.

## Discussion

This scoping review provides a detailed description on the current methodological rigor and overall quality of CPGs specifically designed for use in the out-of-hospital setting. This review identified 15 studies that used four different structured appraisal instruments to assess 311 unique CPGs. CPGs in this review scored poorly against criteria that assessed the methods used to identify, gather, and subsequently appraise evidence. Less than half of the CPGs provided a description of the search strategy used to identify literature or conducted evidence synthesis when assessed against the NAM criteria. Additionally, we observed that the average Domain 3 score for CPGs appraised with the AGREE-II instrument was 56.6%. This domain score assesses the rigor and transparency in which a CPG is developed, and notably, we observed an association between this value and the group category that developed the out-of-hospital guideline. CPGs produced by national institutes or governing bodies and professional medical societies on average, scored more than two-times higher in this domain than CPGs developed by individual ambulance or emergency medical services. This finding aligns with studies from other medical settings that have shown CPGs developed by government-supported organizations score higher than other groups involved in guideline enterprise [36, 37]. To address this issue, future efforts should be directed at increasing collaboration between individual ambulance agencies involved in the provision of out-of-hospital care, as the idiom of ‘many hands make light work’ often rings true. This has been highly successful in the United States, where the Prehospital Guideline Consortium was created in 2016. This group is comprised of various national, state, and local stakeholders and researchers that pool their resources to develop evidence-based CPGs [38]. The creation of similar groups or entities in other geographical locations is recommended.

The publication date of the CPGs included in this review ranged over a 24-year period (1998–2022). We observed no temporal trends in the Domain 3 score over time, which suggests the methodological rigor that underpins out-of-hospital guidelines has not improved and has remained stagnant. This is a concerning finding given the increasing availability of tools, software solutions and training programs that have been designed to assist guideline developers produce robust and transparent CPGs. These include adjuncts such as the GIN-McMaster Guideline Development Checklist, GRADEpro, and INGUIDE [39–41]. We identified that 23.5% of CPGs in this review were reported to be developed in alignment with the GRADE approach, with a significant increase in the use of this framework identified over time. This is an encouraging finding and suggests progressive uptake is occurring however the true utilisation rate of this method by out-of-hospital guideline developers is unknown. We recommend further enquiry is performed to explore and understand this in totality. It can be postulated that by first understanding the methods and approaches currently used by guideline developers that operate in this field, contextualized instruments can be created to ameliorate the rigor in which these CPGs are produced.

The overall quality of CPGs included this review was medium to low with just 34.9% and 0% of the guidelines individually appraised against the NAM and NHMRC criteria meeting all of these standards. In contrast, three of the five CPGs assessed using the JBI Critical Appraisal Tool for Text and Opinion scored equal to or greater than 80%. However, this result is likely spurious given the small sample of CPGs critiqued by this tool. As the AGREE-II instrument does not provide thresholds for what should be considered a ‘high-quality’ CPG, a surrogate definition of equal to or greater than 75% in Domain 3 was used. This definition was based on guidance provided by the authors of this tool in a recent commentary regarding their current practice when endorsing a CPGs for use [23]. Of the 265 CPGs assessed using AGREE-II where this value was completed, just 34.3% met our definition of being high-quality. This is a notable finding given prior literature has reported 90% of CPGs designed for use in the emergency department, and 100% of CPGs used in the orthopedic surgery would be considered high-quality if the same threshold cutoff was used [42, 43]. However, in medicine and healthcare generally, repeated methodological studies have consistently demonstrated lower scores in the rigor of development domain beneath this cutoff [44–46]. As a juvenile discipline of medicine, it is not overly surprising that only one-third of CPGs designed for use in the out-of-hospital setting can be considered high-quality given this field has limited amounts of scientific enquiry. We recommend that guideline developers in this field build on the high-quality CPGs identified in this review by adopting and/or adapting the treatment recommendations contained within these documents into their jurisdiction using evidence-based methodologies such as GRADE-ADOLOPMENT [47, 48]. This development method may reduce evidence-waste which may free guideline developers to create robust and trustworthy CPGs on medical topics where guidance is currently not available.

### Limitations

The findings of this scoping review should be interpreted with acknowledgement of the following limitations. Namely, due to the limitations of this methodology no risk of bias assessment or critical appraisal was performed on the articles that were included. As this review extracted and aggregated information published in other sources, there is a chance that errors may exist in these studies that we cannot account for. Additionally, as the results presented reflect appraised scores, we cannot comment on the veracity of the values reported or the inter-rate reliability. We deliberately did not report ‘the overall guideline assessment’ of CPGs appraised using the AGREE-II instrument given this variable was completed in less than half of the CPGs that were included. In this study, a threshold score of equal to or greater than 75% in Domain 3 was used to categorize CPGs as being high-quality. Other published literature has used a score of 60% in this domain as a threshold which if used in this study, would have resulted in 55.8% (148/65) being deemed as high-quality rather than the 34.4% (91/265) reported.

## Conclusions

This scoping review identified that out-of-hospital CPGs are medium to low quality when assessed by structured appraisal instruments and appear to be developed with poor methodological rigor. The results of this study should be used by guideline developers and academics who operating in this setting to identify new methods and initiatives to improve CPGs.

## Electronic supplementary material

Below is the link to the electronic supplementary material.

**Supplementary Material 1:** Supplementary Material

**Supplementary Material 2:** Appendices

## Data Availability

The authors confirm that the data supporting the findings of this study are available at https://osf.io/z5m7d/files/osfstorage/674961d5881e75bf9b9b6d26.
