# Supplementary material for: Determining the methodological rigor and overall quality of out-of-hospital clinical practice guidelines: a scoping review
Source: Scand J Trauma Resusc Emerg Med. 2025 Feb 21;33:32. doi: 10.1186/s13049-025-01344-z (PMC11846300; doi:10.1186/s13049-025-01344-z)
Supplement: Supplementary file 1 — Supplementary Material 1: Supplementary Material [file 13049_2025_1344_MOESM1_ESM.docx]

**Supplementary Material**

**Supplementary Material 1 – Attempts made to contact authors**

| **Article** | **Email Address Contacted** | **Date of Email** |
| --- | --- | --- |
| Kuupiel D, Jessani NS, Boffa J, Naude C, De Buck E, Vandekerckhove P, McCaul M. Prehospital clinical practice guidelines for unintentional injuries: a scoping review and prioritisation process. BMC Emerg Med. 2023;23(1):27. | - desmondkuupiel98@hotmail.com - dkuupiel@sun.ac.za | 05/06/24  18/06/24  21/08/24 |
| Malherbe P, Smit P, Sharma K, McCaul M. Guidance we can trust? The status and quality of prehospital clinical guidance in sub-Saharan Africa: A scoping review. Afr J Emerg Med. 2021;11(1):79-86. | - malherbepj@gmail.com - pierre.malherbe@ochsner.org | 05/06/24  18/06/24 |
| Yousefifard M, Askarian-Amiri S, Madani Neishaboori A, Sadeghi M, Saberian P, Baratloo A. Pre-hospital pain management; a systematic review of proposed guidelines. Arch Acad Emerg Med. 2019;7(1):e55. | - m_nasr54@med.mui.ac.ir | 05/06/24  18/06/24 |
| Muntlin Athlin Å, Juhlin C, Jangland E. Lack of existing guidelines for a large group of patients in Sweden: a national survey across the acute surgical care delivery chain. J Eval Clin Pract. 2017;23(1):89-95. | - asa.muntlin@pubcare.uu.se | 05/06/24  18/06/24 |

**Supplementary Material 2 – Extraction tool and raw data**

The extraction tool and raw data reported in this study are available at the following online repository https://osf.io/z5m7d/files/osfstorage/674961d5881e75bf9b9b6d26

**Supplementary Material 3 – Articles that were excluded from review.**

| **Title** | **Reason for Exclusion** |
| --- | --- |
| Gage, C. B., Powell, J. R., Bosson, N., Crowe, R., Guild, K., Yeung, M., … Panchal, A. R. (2023). Evidence-Based Guidelines for Prehospital Airway Management: Methods and Resources Document. Prehospital Emergency Care, 28(4), 561–567. https://doi.org/10.1080/10903127.2023.2281377 | Did not appraise CPGs |
| Hawryluk, Gregory W. J. MD, PhD*,‡; Lulla, Al MD§; Bell, Randy MD‖; Jagoda, Andy MD¶; Mangat, Halinder S. MD, MSc, FNCS, FCCM‡,**; Bobrow, Bentley J. MD‡‡; Ghajar, Jamshid MD, PhD‡. Guidelines for Prehospital Management of Traumatic Brain Injury 3rd Edition: Executive Summary. Neurosurgery 93(6):p e159-e169, December 2023. \| DOI: 10.1227/neu.0000000000002672 | Did not appraise CPGs |
| Andrew F Whyte, Jasmeet Soar, Amy Dodd, Anna Hughes, Nicholas Sargant, Paul J Turner, Emergency treatment of anaphylaxis: concise clinical guidance, Clinical Medicine, Volume 22, Issue 4, 2022, Pages 332-339, ISSN 1470-2118, https://doi.org/10.7861/clinmed.2022-0073. | Did not appraise CPGs |
| Martin-Gill, C., Panchal, A. R., Cash, R. E., Richards, C. T., Brown, K. M., & Patterson, P. D. (2022). Recommendations for Improving the Quality of Prehospital Evidence-Based Guidelines. Prehospital Emergency Care, 27(2), 121–130. https://doi.org/10.1080/10903127.2022.2142992 | Did not appraise CPGs |
| Thomson, Michelle1; Simpson, Paul2; Pap, Robin2; Munn, Zachary1. Clinical practice guidelines in prehospital pain management for paramedics: a systematic review protocol. JBI Evidence Synthesis 22(3):p 461-471, March 2024. \| DOI: 10.11124/JBIES-23-00128 | Review protocol |
| Fouche PF, Zverinova K. An Appraisal of the Australian Resuscitation Council Arrhythmia Guideline. Australasian Journal of Paramedicine. 2014;11:1-7. | Wrong context (in-hospital setting) |
