# Supplementary material for: Determining the methodological rigor and overall quality of out-of-hospital clinical practice guidelines: a scoping review
Source: Scand J Trauma Resusc Emerg Med. 2025 Feb 21;33:32. doi: 10.1186/s13049-025-01344-z (PMC11846300; doi:10.1186/s13049-025-01344-z)
Supplement: Supplementary file 2 — Supplementary Material 2: Appendices [file 13049_2025_1344_MOESM2_ESM.docx]

**Appendices**

**Appendix 1 – Search strategy**

**MEDLINE (Ovid) Search Strategy**

| **#** | **Search** | **No. Results Retrieved** |
| --- | --- | --- |
| 1 | (Ambulance or Emergency Medical Service).sh. or ("out-of-hospital" or prehospital or pre-hospital or paramedic* or ambulance).ti,ab,kf. | 50,761 |
| 2 | (Practice Guideline or Evidence-Based Medicine).sh or (guideline* or clinical practice guideline* or evidence-based guideline* or recommendation* or policy).ti,ab,kf. | 1,101,299 |
| 3 | (Appraisal or "AGREE II" or systematic review or guideline quality or content analysis or document analysis).ti,ab,kf. | 384,980 |
| 4 | #1 AND #2 AND #3 | 527 |

Ti = Title

AB = Abstract

KF = Keyword Heading Word

**EMBASE (Elsevier) Search Strategy**

| **#** | **Search** | **No. Results Retrieved** |
| --- | --- | --- |
| 1 | (Ambulance or Emergency Medical Service).sh. or ("out-of-hospital" or prehospital or pre-hospital or paramedic* or ambulance).ti,ab,kf. | 80,424 |
| 2 | (Practice Guideline or Evidence-Based Medicine).sh or (guideline* or clinical practice guideline* or evidence-based guideline* or recommendation* or policy).ti,ab,kf. | 1,769,944 |
| 3 | (Appraisal or "AGREE II" or systematic review or guideline quality or content analysis or document analysis).ti,ab,kf. | 459,641 |
| 4 | #1 AND #2 AND #3 | 637 |

**CINAHL Search Strategy**

| **Search** | **No. Results Retrieved** |
| --- | --- |
| ( (Ambulance or Emergency Medical Service) or ("out-of-hospital" or prehospital or pre-hospital or paramedic* or ambulance) ) AND ( (Practice Guideline or Evidence-Based Medicine).sh or (guideline* or clinical practice guideline* or evidence-based guideline* or recommendation* or policy) ) AND ( (Appraisal or "AGREE II" or systematic review or guideline quality or content analysis or document analysis) ) | 1,712 |

**SCOPUS Search Strategy**

| **Search** | **No. Results Retrieved** |
| --- | --- |
| ( TITLE-ABS-KEY ( ambulance OR "emergency medical service" OR "out of hospital" OR prehospital OR paramedic* ) AND TITLE-ABS-KEY ( "practice guideline" OR "evidence-based medicine" OR guideline* OR "clinical practice guideline*" OR "evidence-based guideline*" OR recommendation* OR policy ) AND TITLE-ABS-KEY ( appraisal OR "AGREE II" OR "systematic review" OR "guideline quality" OR "content analysis" OR "document analysis" ) ) | 1,337 |

**Proquest Search Strategy**

| **Search** | **No. Results Retrieved** |
| --- | --- |
| (ambulance OR "emergency medical service" OR "out of hospital" OR prehospital OR paramedic*) AND noft(("practice guideline" OR "evidence-based medicine" OR guideline* OR "clinical practice guideline*" OR "evidence-based guideline*" OR recommendation* OR policy)) AND noft((appraisal OR "AGREE II" OR "systematic review" OR "guideline quality" OR "content analysis" OR "document analysis")) | 1,546 |
